# Enhancing Data Freshness in Air-Ground Collaborative Heterogeneous Networks through Contract Theory and Generative Diffusion-Based Mobile Edge Computing

**DOI:** 10.3390/s24010074

**Published:** 2023-12-22

**Authors:** Zhiyao Sun, Guifen Chen

**Affiliations:** School of Electronic Information Engineering, Changchun University of Science and Technology, Changchun 130000, China

**Keywords:** air-ground collaborative heterogeneous networks, age of information, contract theory, generative diffusion model, mobile edge computing

## Abstract

Mobile edge computing is critical for improving the user experience of latency-sensitive and freshness-based applications. This paper provides insights into the potential of non-orthogonal multiple access (NOMA) convergence with heterogeneous air–ground collaborative networks to improve system throughput and spectral efficiency. Coordinated resource allocation between UAVs and MEC servers, especially in the NOMA framework, is addressed as a key challenge. Under the unrealistic assumption that edge nodes contribute resources indiscriminately, we introduce a two-stage incentive mechanism. The model is based on contract theory and aims at optimizing the utility of the service provider (SP) under the constraints of individual rationality (IR) and incentive compatibility (IC) of the mobile user. The block coordinate descent method is used to refine the contract design and complemented by a generative diffusion model to improve the efficiency of searching for contracts. During the deployment process, the study emphasizes the positioning of UAVs to maximize SP effectiveness. An improved differential evolutionary algorithm is introduced to optimize the positioning of UAVs. Extensive evaluation shows our approach has excellent effectiveness and robustness in deterministic and unpredictable scenarios.

## 1. Introduction

The generation of mobile data is growing at an unprecedented rate due to the boom in Internet of Things (IoT) sensors and mobile technologies. This development has led to an influx of mobile tasks that are latency sensitive and computationally intensive, thus highlighting the need for efficient processing mechanisms. Mobile edge computing (MEC) is a new paradigm of innovation. The idea of MEC, which provides computing and storing capabilities directly within the wireless access network, is described in detail in [1]. In addition to reducing congestion in the centralized network, MECs reduce the associated costs, particularly those associated with latency and power consumption for mobile tasks, by offloading the processing of tasks to the edge nodes. Understandably, this has made MECs the center of academic interest, which has spawned a large number of research efforts aimed at refining task offload determination and resource allocation techniques to improve the execution of mobile tasks [2]. However, the great potential of MEC is not without its challenges. In particular, it is constrained by limited computational power and limited spectrum resources. The age of information (AoI) serves as a metric that elegantly quantifies the temporal freshness of data by indicating the length of time since the last update was released. Task offloading decisions that are influenced by the AoI are critical to ensuring the timeliness and relevance of the computation and data processing [3]. Thus, the shortcomings described above are particularly evident for computational tasks that are closely associated with the information age.

With the rapid development of MEC technology, real-time applications have expanded significantly. These include remote monitoring, disaster mitigation, smart agriculture, and urban logistics. There has been a promising integration of unmanned aerial vehicles (UAVs) and non-orthogonal multiple access (NOMA) in the technology domain to address the inherent limitations of multi-spectrum access technologies. This convergence has resulted in an air–ground cooperative heterogeneous network (AGCHN) model [4]. The proposed model not only characterizes the complexity of heterogeneous networks in terms of homogeneous and cross-layer interference, but also explores collaborative research on computational task offloading and resource allocation for HetNet and MEC interconnections [5,6,7,8]. This model is strategically designed to optimize MEC throughput and spectrum resource utilization. In particular, the integration of UAVs has the goal of compensating for the computational limitations of MECs, thus strengthening their computational framework. At the same time, the emergence of NOMA will play a key role in ensuring the maximum utilization of the spectrum resources, thus improving the overall efficiency of the system.

Indeed, the seamless integration of UAVs and NOMA into the MEC architecture is a delicate challenge. This challenge is compounded by the requirements of AoI-based mission offload. The synergy of these technologies is critical to improving the functional efficiency of the MEC, and a judicious mix of resource optimization schemes is required to support AoI-driven mission offloading [4,9]. In practical situations, it is clear that the edge devices will not allocate resources without sufficient incentives. From this operational perspective, using advanced incentivizing schemes becomes particularly important [10,11]. The utilization of artificial intelligence has facilitated the creation of adaptable systems for effectively addressing novel contexts. The utilization of deep learning and deep reinforcement learning techniques accounts for this phenomenon [12]. It is possible to motivate and ensure that these edge nodes contribute resources consistently and optimally by designing well thought-out and tailored incentive mechanisms [13].

The following is a list of the main contributions in this paper.

- This study designs an incentive mechanism that includes both the contract design and the deployment phases. In particular, during the contract design phase, we model the quality of service of users as a function of delay and age of the information, taking into account NOMA communication. Based on this model, we then design the incentive mechanism to account for asymmetric information.
- During the contract design phase, we utilize contract theory to model a contract problem that optimizes the utility of a service provider (SP) while satisfying individual rationality (IR) and incentive compatibility (IC) criteria for mobile users. By reducing constraints and transforming variables, the original contract problem is transformed into a problem that can be solved by the BCD algorithm. To enhance the effectiveness of finding the optimal contract, a new solution based on the generative diffusion mode is proposed.
- During the contract deployment phase, we strive to maximize the utility of the SP contract deployment by optimizing the location of the UAV. To this end, an improved differential evolution algorithm is proposed to identify the optimal UAV locations.
- Compared with the existing methods, our numerical analysis experiment proves that our proposed methods are better and more effective under certain and uncertain environments.

The rest of this paper is structured as follows. The related work is presented in Section 2. The system model and problem formulation are presented in Section 3. The problem formulation is presented in Section 4. Section 5 shows performance evaluation. Section 6 concludes this paper.

## 2. Related Work

The domain of MEC has garnered substantial attention within the mobile communication networks realm [14]. This innovation offers a promising avenue to circumvent the constraints of conventional centralized cloud computing by propelling computing and storage resources proximate to end users. The integration of MEC capabilities strategically optimizes offload policies for mobile devices, ushering in an era of elevated service quality and minimal latency in data transmission.

At the same time, AoI has become an important parameter to measure the freshness of data in communication systems. It has gained attention in several applications, such as real-time control, smart agriculture, and healthcare, especially modeling the age problem in wireless networks [15,16]. This has spawned innovative indexing methods and led to intensive research on how to optimize AoI during offloading to improve real-time performance in mobile networks.

Within the sphere of MEC networks, Kuang et al. provided seminal insights into AoI by examining an MEC network model encompassing a single source and server. They arrived at a distinct expression for average AoI and assessed the implications of different MEC offloading strategies [17]. Following this, a study advanced the discussion by analyzing AoI under a partial offloading strategy, emphasizing the significance of optimal offloading ratios [18].

Liu et al. innovatively tackled the challenge of AoI in multi-source, single-server MEC systems by introducing a novel online algorithm that optimally manages task generation, computation offload, and resource distribution [19]. In the realm of MEC-facilitated IoT, Muhammad et al. analyzed the interplay of MEC and AoI, highlighting the pivotal role of variables such as random arrivals and unstable channel conditions [20]. Zhu et al. designed an online optimization algorithm, leveraging both Lyapunov and convex optimization techniques, to ensure optimal online decision making even under partially stale network conditions [21].

In wireless sensor networks (WSNs), Zhang et al. [22] wrestled with AoI-aware scheduling. They jointly optimized sampling rate, computing schedule, and transmit power, tackling a non-convex average AoI minimization problem using geometric programming and sequential convex approximation techniques.

In the context of UAV-aided IoT networks and to quantify the freshness of information, AoI has been widely used in some recent work. Reference [23] in UAV-enabled edge computing focuses on optimizing computational offloading. Employing a Stackelberg multi-layer game, the study aligns interests of base stations, drones, and users, emphasizing time delay and energy consumption. Simulations underscore the Stackelberg game algorithm (SGA) as surpassing conventional methods. Chen et al. [24] delve into AoI perceptual resource management within UAV-assisted mobile electronic device systems orchestrated by an infrastructure provider (InP). Highlighting non-cooperative dynamics, their work models interactions as a stochastic game, with mobile users (MUs) vying for constrained resources. The researchers pioneer an online deep reinforcement learning (DRL) methodology, deploying dual deep Q-networks for individual MUs. Numerical evaluations underscore the DRL’s prowess in harmonizing AoI with energy utilization. Recent exploration ventured into the air–ground collaborative mobile edge computing (AGC-MEC) network, wherein Qin et al. [25] jointly optimized task scheduling, computational resource allocation, and unmanned aerial vehicle (UAV) trajectory to reduce ground user devices’ (UEs) weighted AoI. The approach dynamically tailored offloading design, safeguarding computational output freshness, while optimizing scheduling to enhance performance.

As NOMA rises to prominence as a method to amplify network capacity, integrating it with MEC has spurred a wealth of research. Zhu and Liu both offered insights into this integration, with Zhu emphasizing spectrum utilization in a single-user multi-edge server MEC system [26] and Liu scrutinizing the AoI performance of a NOMA-informed MEC offloading strategy [27].

Recent literature also underscores various approaches to mobile device offloading in NOMA-anchored MEC networks. For instance, game theory has been a popular tool, and Yang et al. addresses channel access issues through the ordinary potential game [28]. The study by Lv et al. highlights the criticality of resource constraints in edge computing, proposing a novel online auction mechanism [29].

Recent trends highlight the growing role of contract theory in wireless networks [30]. This theory aids in crafting incentives to motivate user participation in activities like mobile offloading. Its application promises better network performance and equitable user rewards. Lim et al. proposed a contract-theoretic scheme, taking into account varying AoI requirements across tasks [31]. This method adapts to diverse AoI and latency preferences, as confirmed by experiments.

Recently, reinforcement learning has been used by certain academics to address the challenge of mobile edge computing resource optimization, strengthening the effectiveness of the incentive mechanism. Li et al. harnessed DRL and game theory for computation offloading in MEC systems [32]. Their strategy, built on proximal policy optimization (PPO), optimizes data offload size, pricing, and MEC server selection. This results in enhanced stability and faster convergence in simulations. Xu et al. focused on incentive design for mobile crowd sensing, integrating the AoI metric for data freshness [33]. Their two-tiered Stackelberg game-based approach offers AoI-centric incentives and a DRL-driven method for uncertain utility parameters. Both strategies demonstrate effectiveness in real-world tests.

The research arena of mobile edge computing task offloading with an emphasis on AoI is emerging. Notably, some scholars have ventured into joint optimizations of AoI with other critical performance indicators, including delay and energy consumption. Nevertheless, the prevalent trend in this research is a systemic optimization approach, with limited exploration from the perspective of incentive mechanisms. Furthermore, an evident oversight in current studies, as highlighted in Table 1, is the disregard for interference dynamics in air–ground cooperative heterogeneous network settings. Efforts to design optimal offloading strategies for mobile devices in air–ground collaboration scenarios utilizing NOMA, contract theory, and MEC do face some inherent challenges. These challenges include the AoI variability of mission information, different user requirements, and the complexity associated with contract design. Overall, explorting AoI in conjunction with reinforcement learning to augment heterogeneous networks for air–ground cooperation, particularly through NOMA and MEC offloading frameworks infused with contract theory, is still a nascent field. Undoubtedly, this area requires deeper and broader research efforts.

## 3. System Model

Section 3 describes the underlying framework of the study. Section 3.1 details the utility of mobile terminals (MT). Section 3.2 describes the service provider’s utility. Section 3.3, Contract Formulation, describes the methodology for reaching agreements between stakeholders. Together, these subsections provide a concise overview of system operation, stakeholder interests, and contract strategies.

### 3.1. Utility of Mobile Terminal

**Figure 1:**
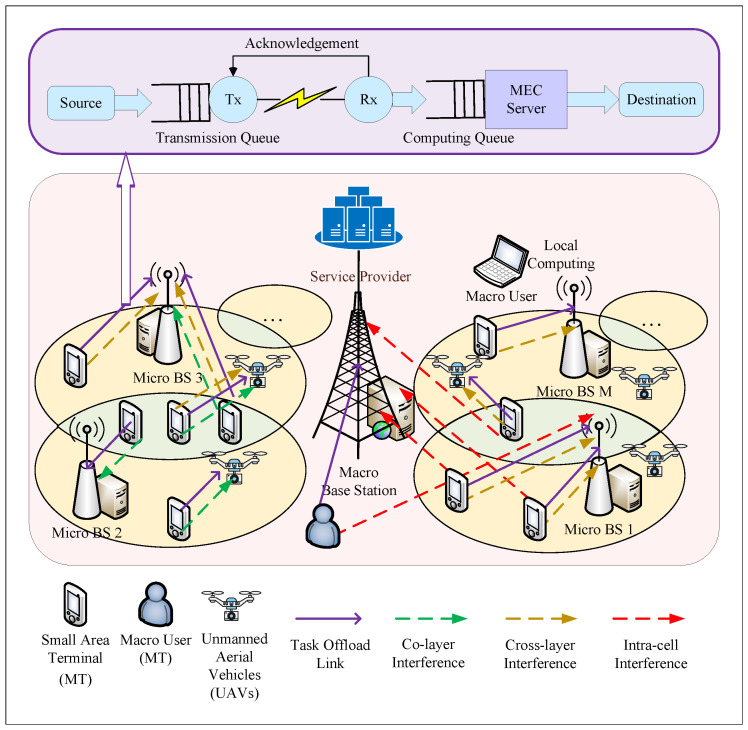
Heterogeneous network model (HNM).

In the presence of a single SP, we depict in Figure 1 an air–ground cooperative heterogeneous network model in which ground-based MEC servers and UAVs jointly provide computational power at the network edge. The network consists of a macro base station, *M* micro base stations containing MEC servers, macro users, and *V* UAVs. A device served by the MEC server *m* is defined as $n_{m,v}$, where v=0. Each MEC server m∈M is connected to a group of $N_{m,v}$. A device served by the UAV $v_{m}$ in the MEC server *m* is defined as $n_{m,v}$, where $1\leq v\leq V_{m}$. Each UAV $v_{m}\in V_{m}$ is connected to a group of $N_{m,v}$. For ease of reference, Table 2 summarizes the key notations.

The SP and MEC servers are connected by high-speed fiber optic cables for reliable communication. The MT interacts with the MEC servers using non-orthogonal NOMA technology over wireless channels. Each UAV functions as a dedicated parallel computing server, which boosts the network’s computational prowess and responsiveness. In this instance, factors including intra-cell interference, cross-layer interference, and co-layer interference must be taken into account.

Figure 1 shows a dual-node cascaded model with two cascaded queues: the transmission queue and the MEC computation queue.

Assuming that the service times of both queues follow an exponential distribution, with service rates denoted as $\mu_{m,v,n}^{t}$ and $\mu_{m,v,n}^{s}$, respectively, the queue utilization for $n_{m,v}$ for edge node (MEC server *m* or UAV $v_{m}$) is denoted by $\rho_{m,v,n}$

(1) $$\rho_{m,v,n}=\frac{\mu_{m,v,n}^{t}}{\mu_{m,v,n}^{s}}$$

In order to maintain queue stability, it is stipulated $\rho_{m,v,n}<1$ and $\rho_{m,v,n}<1$.

When unloading, MTs offload computing tasks that require δ (Mcycles) CPU cycles and have a data packet size of *s* (MBits) through a wireless channel to an edge node with a computational capability of $f_{m,v,n}$. Let $h_{m,v,n}$ represent the channel power gain between the edge node and MT $n_{m,v}$.

Referring to [34], $h_{m,v,n}$ is defined as $h_{m,v,n}=\varphi \;{\left\|l_{m,v}-l_{m,v,n}\right\|}^{-2}$, where φ represents the channel power at the reference distance, $l_{m,v}$ is the location of the MEC server *m* or UAV $v_{m}$, and $l_{m,v,n}$ is the location of MT $n_{m,v}$. Let $d_{m,v,n}$ denote the MTs’ transmission power and *B* denote the available system bandwidth. Therefore, when MT $n_{m,v}$ uploads its computational task to an edge node, we adopt NOMA [35] technology to define the achievable transmission rate

(2) $$\begin{array}{c} R_{m,v,n}=B\log_{2}\left(1+\frac{d_{m,v,n}h_{m,v,n}}{\zeta +\sum_{m\in M}\sum_{v\in V_{m}}\sum_{j\in N_{m,v},h_{m,v,j}\geq h_{m,v,n}}d_{m,v,j}h_{m,v,j}}\right) \end{array}$$

where ζ is the power spectral density of Gaussian white noise and $V_{m}=1$ with v=0. Thus, for the transfer queue, its service rate $\mu_{m,v,n}^{t}$ can be rewritten as

(3) $$\mu_{m,v,n}^{t}=\frac{R_{m,v,n}}{s}$$

For the computation queue, its service rate $\mu_{m}^{s}$ can be rewritten as

(4) $$\mu_{m,v,n}^{s}=\frac{f}{\delta }$$

Referring to [18] and based on (3) and (4), the average AoI expression for MT $n_{m,v}$ is defined as

(5) $$A_{m,v,n}=\frac{1}{\mu^{s}}\left[\frac{\rho_{m,v,n}\left(2\rho_{m,v,n}^{2}-\rho_{m,v,n}+1\right)}{1-\rho_{m,v,n}^{2}}+\frac{2}{\rho_{m,v,n}}+1\right]$$

In addition, the average service delay is defined as

(6) $$T_{m,v,n}=\frac{1}{\mu_{m,v,n}^{s}}+\frac{1}{\mu_{m,v,n}^{t}}$$

Referring to [36], the gain obtained is defined as the saved delay, and AoI by unloading the task of MT $n_{m,v}$ is

(7) $$g\left(d_{m,v}\right)=\psi \left(T^{\max}-T_{m,v,n}\right)+\left(1-\psi \right)\left(A^{\max}-A_{m,v,n}\right)$$

where $A_{max}$ is the maximum tolerated AoI and ψ is the trade-off parameter between the saved delay and saved AOI. The utility of MT $n_{m,v}$ is the difference between the gained satisfaction and its cost $p_{m,v,n}$ and $p_{m,v,n}^{v}$ of offloading its task to the SP or UAV. Thus, the utility of MT $n_{m,v}$ is

(8) $$u_{m,v}=\theta_{m,v,n}g\left(d_{m,v,n}\right)-p_{m,v,n}$$

where $\theta_{m,v,n}$ is the satisfaction of the performance of the saved delay.

It is noted that the $N_{m,v}$ MTs’ type belonging to their privacy is not visible to edge nodes, namely information asymmetry. Various satisfaction categories can be used to categorize $N_{m,v}$ MTs according to their composition. These are the definitions that apply. The set of variables $\Theta_{m,v}$ is defined as the collection of $\Theta_{m,v}$ for every *i* such that $\left\{\theta_{m,v,i}:1\leq i\leq I_{m,v}\right\}$. Consequently, the number of classes of MTs can be denoted as $I_{m,v}$. The probability distribution for each category is denoted as $q_{m,v,i}$, where *m*, *v*, and *i* represent specific indices. The corresponding number for each category is given by the product of $N_{m,v}$ and $q_{m,v,i}$, that is, $N_{m,v}q_{m,v,i}$. In other words, the sum of all the products $N_{m,v}q_{m,v,i}$ over the set of indices *i* in $I_{m,v}$ is equal to $N_{m,v}$. Sequences of MTs that are not degenerate are organized based on their kind.

(9) $$\begin{array}{c} 0<\theta_{m,v,1}\leq \theta_{m,v,2}\leq \cdots \leq \theta_{m,v,I} \end{array}$$

A higher θ indicates the higher satisfaction of the performance of the computation task. In this case, $\left(d_{m,v,i},p_{m,v,i}\right)$ is the contract for MTs of type (m,v,i). Each edge node will offer different contracts based on the θ of the MTs instead of offering the same contract to all of the MTs. We assume that the MT signs a contract of (0,0) if the MT rejects any contract.

The utility of a MT of type (m,v,i) is then redefined as follows:

(10) $$\begin{array}{c} u_{m,v,i}=\theta_{m,v,i}g\left(d_{m,v,i}\right)-p_{m,v,i}. \end{array}$$

### 3.2. Utility of Service Provider

The expense to finish the computation task that the MT offloaded is borne by the MEC service provider. Referring to [37], in order to coordinate with other MTs to lessen the effects of interference and to enable the MT to send data with $d_{m,v,i}$ power, the SP pays a unit cost of $c_{1}^{m,v}$ for these offloaded computation tasks, i.e., the cost is $c_{1}^{m,v}d_{m,v,i}$. Referring to [38], the cost of computing to complete a task is denoted by the formula $c_{2}^{m,v}\eta \kappa_{m,v}f_{m,v}^{2}$, where $c_{2}^{m,v}$ denotes the unit cost expenditure for computational energy consumption and $\kappa_{m,v}$ is an effective switching capacitor. Thus, the utility obtained by the edge node is as follows:

(11) $$\begin{array}{c} U_{m,v,i}=p_{m,v,i}-c_{1}^{m,v}d_{m,v,i}-c_{2}^{m,v}\eta_{m,v}\kappa_{m,v}f_{m,v}^{2} \end{array}$$

As a result, the utility of the SP is as follows for all types of MTs:

(12) $$\begin{array}{c} U_{\mathrm{sp}}=\sum_{m\in M}\sum_{v\in V_{m}}\sum_{i\in I_{m,v}}N_{m,v}q_{m,v,i}U_{m,v,i} \end{array}$$

To simply the expression, we define the unit transmission rate as the following:

(13) $$\begin{array}{c} r_{m,v,i}=\frac{R_{m,v,i}}{B} \end{array}$$

Furthermore, based on (3) and (4), we have

(14) $$\rho_{m,v,i}=\frac{\mu_{m,v,i}^{t}}{\mu^{s}}=\frac{Br_{m,v,i}\delta }{sf}=r_{m,v,i}\eta$$

where $\eta =\frac{B\delta }{sf}$. When $d_{m,v,j}\forall j\in I,j\neq i,m\in M$ is fixed, $r_{m,v,i}$ is monotonically increasing in terms of $d_{m,v,i}$, which indicates that $r_{m,v,i}$ and $d_{m,v,i}$ have one-to-one correspondence. For the sake of later analysis, we replace $d_{m,v,i}$ with $r_{m,v,i}$ in (10) and obtain

(15) $$\begin{array}{c} u_{m,v,i}=\theta_{m,v,i}g\left(r_{m,v,i}\right)-p_{m,v,i} \end{array}$$

Then, the SP’s utility is rewritten as

(16) $$\begin{array}{c} U_{\mathrm{sp}}=\sum_{m\in M}\sum_{v\in V_{m}}\sum_{i\in I_{m,v}}N_{m,v}q_{m,v,i}\left(p_{m,v,i}-c_{1}^{m,v}d_{m,v,i}\left(r\right)-c_{2}^{m,v}\eta_{m,v}\kappa_{m,v}f_{m,v}^{2}\right), \end{array}$$

where $d_{m,v,i}\left(r\right)=\frac{\left(2^{r_{m,v,i}}-1\right)\left(\zeta +\sum_{m\in M}\sum_{v\in V_{m}}\sum_{j\in N_{m,v},h_{m,v,j}\geq h_{m,v,n}}N_{m,v}q_{m,v,j}d_{m,v,j}h_{m,v,j}\right)}{h_{m,v,i}}$,

$r=[r_{1},r_{2},\cdots ,r_{M}]$, $r_{m}=\left[r_{m,1},r_{m,2},\cdots ,r_{m,V}\right]$ and $r_{m,v}=\left[r_{m,v,1},r_{m,v,2},\cdots ,r_{m,v,I}\right]$.

### 3.3. Contract Formulation

It is noted that the MTs’ type belonging to their privacy is not visible to the SP, namely information asymmetry. Considering this information asymmetry, the SP can apply contract theory to find out the best MTs. Here, the SP is the principal of the design contract, and the MTs are agents who select the contract item fitting their own corresponding type. The contract item can be denoted as $\Phi =\left\{\left(r_{m,v,i},p_{m,v,i}\right),i\in I_{m,v},v\in V_{m},m\in M\right\}$, where $\left(r_{m,i},p_{m,i}\right)$ is made for type-$\theta_{m,i}$ MTs. Under the two-tuple asymmetric information, we introduce the following IR conditions and IC conditions to design a feasible contract. The IR condition encourages the participation of MTs while ensuring the non-negative utility of the MT that participates in the computation task. Thus, the IR conditions for the MT of type-$\theta_{m,v,i}$ will be

(17) $$\begin{array}{c} u_{m,v,i}\geq 0,i\in I_{m,v},v\in V_{m},m\in M \end{array}$$

The IC conditions ensure that each type of MT can achieve its maximum utility when selecting the contract item based on its own corresponding type. In other words, any item $\left(r_{m,v,i},p_{m,v,i}\right)$, $\forall i\in I_{m,v}$ cannot maximize the utility of type-$\theta_{m,v,i}$ MT except the contract item $\left(r_{m,v,i},p_{m,v,i}\right)$.

Thus, the IC conditions for type-$\theta_{m,v,i}$ MT can be denoted as

(18) $$\begin{array}{c} \theta_{m,v,i}g\left(r_{m,v,i}\right)-p_{m,v,i}\geq \theta_{m,v,i}g\left(r_{m,v,j}\right)-p_{m,v,j},\forall i,j\in I_{m,v},v\in V_{m},m\in M \end{array}$$

Our objective is to maximize the utility of the SP under IR and IC constraints, which is formulated into a contract optimization problem as

**Problem** **1.**
(19) $$\begin{array}{cc} \; & \;\max_{r_{m,v,i},p_{m,v,i}}U_{\mathrm{sp}}\\ s.t. & C1:\;\left(17\right),i\in I_{m,v},v\in V_{m},m\in M.\\ \; & C2:\;\left(18\right),i\in I_{m,v},v\in V_{m},m\in M. \end{array}$$

In the next section, we will simplify the constraints in the optimization problem and provide an analysis for solving the simplified optimization problem.

## 4. Optimal Contract Design and Contract Deployment

We begin by exploring mathematical contract design in Section 4.1 of this section. In Section 4.2, AI-generated contract design introduces an AI-centric approach. Finally, in Section 4.3, we emphasize the practicality of using contracts in the real world.

### 4.1. Mathematical Based Contract Design

Since there are $\sum_{m\in M}\sum_{v\in V_{m}}N_{m,v}$ IR constraints and $\sum_{m\in M}\sum_{v\in V_{m}}N_{m,v}\left(N_{m,v}-1\right)$ IC constraints in Problem 1, it is difficult to directly solve Problem 1 with complicated constraints. Specifically, it refers to the challenges posed by the large number of constraints associated with the increasing number of contracts. As the number of contracts increases, the constraints grow exponentially, and their non-convexity increases the complexity of the formula. Thus, we first reduce the number of attached constraints to reformulate Problem 1. Then, we further derive the solution theoretically.

(20) $$\begin{array}{cc} C1: & \theta_{m,v,1}g\left(r_{m,v,1}\right)-p_{m,v,1}\geq 0,\\ C2: & \theta_{m,v,i}g\left(r_{m,v,i}\right)-p_{m,v,i}\geq \theta_{m,v,i}g\left(r_{m,v,i-1}\right)-p_{m,v,i-1},\;\forall i\in \left\{2,\ldots ,I_{m,v}\right\},\\ C3: & \theta_{m,v,i}g\left(r_{m,v,i}\right)-p_{m,v,i}\geq \theta_{m,v,i}g\left(r_{m,v,i+1}\right)-p_{m,v,i+1},\;\forall i\in \left\{1,\ldots ,I_{m,v}-1\right\},\\ C4: & 0\leq r_{m,v,1}\leq r_{m,v,2}\leq \cdots \leq r_{m,v,I},\;0\leq p_{m,v,1}\leq p_{m,v,2}\leq \cdots \leq p_{m,v,I}. \end{array}$$

**Proof.**  Please refer to [39].    □

Constraint (**C1**) related to the IR constraints ensures that the utility of each worker receiving the contract item of its type is non-negative. Constraints (**C2**), (**C3**), and (**C4**) are related to the IC constraints. Constraints (**C2**) and (**C3**) show that the IC constraints can be reduced as local downward incentive compatibility (LDIC) and local upward incentive compatibility (LUIC) with monotonicity, respectively [40]. Constraint (**C4**) indicates that a worker type with a lower cost can provide the SP with a higher update frequency. From Equation (20), we can know that when the lowest-type workers satisfy the IR constraints, the other types of workers will automatically hold the IR constraints. When type-(m,v,i) and type-(m,v,i−1) workers satisfy the IC constraints, the type-(m,v,i) and the other types of workers will automatically hold the IC constraints. Thus, the original $\sum_{m\in M}\sum_{v\in V_{m}}I_{m,v}^{2}$ IR and IC constraints are transformed into $\sum_{m\in M}\sum_{v\in V_{m}}\left(I_{m,v}+1\right)$ constraints, and **Problem 1** can be reformulated as

**Problem** **2.**
(21) $$\begin{array}{cc} \; & \;\max_{\left\{\left(r_{m,v,i},p_{m,v,i}\right)\right\}}U_{\mathrm{sp}}\\ s.t.C1:\; & \theta_{m,v,1}g\left(r_{m,v,1}\right)-p_{m,v,1}=0,\\ C2:\; & \theta_{m,v,i}g\left(r_{m,v,i}\right)-p_{m,v,i}=\theta_{m,v,i}g\left(r_{m,v,i-1}\right)-p_{m,v,i-1},i\in I_{m,v},v\in V_{m},m\in M,\\ C3:\; & r_{m,v,i}\geq 0,i\in I_{m,v},v\in V_{m},m\in M,\\ C4:\; & p_{m,v,i}\geq 0,i\in I_{m,v},v\in V_{m},m\in M. \end{array}$$

Based on the first two constraints of (21), the optimal reward $p_{m,v,i}^{*}$ is calculated by the iterative method in a subsequent way. Thus, we can obtain $p_{m,v,i}^{*}$ as

(22) $$\begin{array}{c} p_{m,v,i}=\sum_{z=1}^{i}\Delta_{m,v,z}+\theta_{m,v,1}g\left(r_{m,v,1}\right) \end{array}$$

where $\Delta_{m,v,1}=0$, $\Delta_{m,v,z}=\theta_{m,v,z}g\left(r_{m,v,z}\right)-\theta_{m,v,z}g\left(r_{m,v,z-1}\right),z\in Z,i\in I_{m,v},v\in V_{m},m\in M$.

By substituting (22) into $U_{sp}$ in **Problem 1**, **Problem 2** is reformulated as

**Problem** **3.** (23) $$\begin{array}{cc} \; & \;\max_{\left\{r_{m,v,i}\right\}}U_{\mathrm{sp}}=\sum_{m\in M}\sum_{v\in V_{m}}\sum_{i\in I_{m,v}}U_{m,v,i}\\ s.t.C1:\; & r_{m,v,i}\geq 0,i\in I_{m,v},v\in V_{m},m\in M. \end{array}$$

where

$$U_{m,v,i}=\left\{\begin{array}{c} \theta_{m,v,i}g\left(r_{m,v,i}\right)\sum_{a=i}^{I}N_{m,v}q_{m,v,a}\\ -\theta_{m,v,i+1}g\left(r_{m,v,i}\right)\sum_{b=i+1}^{I}N_{m,v}q_{m,v,b}-N_{m,v}q_{m,v,i}c_{1}^{m,v}d_{m,v,i}\left(r\right)\\ -N_{m,v}q_{m,v,i}c_{2}^{m,v}\eta_{m,v}\kappa_{m,v}f_{m,v}^{2},0<i<I_{m,v}\\ N_{m,v}q_{m,v,i}\theta_{m,v,i}g\left(r_{m,v,i}\right)-N_{m,v}q_{m,v,i}c_{1}^{m,v}d_{m,v,i}\left(r\right)\\ -N_{m,v}q_{m,v,i}c_{2}^{m,v}\eta \kappa_{m,v}f_{m,v}^{2},i=I_{m,v}. \end{array}\right.$$

Since the optimization variables are coupled together, the functions and constraints are non-convex.

When $R_{-m,v,i}$ is fixed and $r_{m,v,i}<\frac{1}{\eta },\forall m\in M,\forall i\in I$, the following **Problem 4** is a convex problem in terms of $r_{m,v,i}$. **Problem 4** is defined as the following:

**Problem** **4.** (24) $$\begin{array}{cc} \; & \;\min_{r}U_{\mathrm{sp}}^{{}^{\prime }}=-\sum_{m\in M}\sum_{v\in V_{m}}\sum_{i\in I_{m,v}}U_{m,v,i}\\ s.t.C1:\; & r_{m,v,i}\geq 0,i\in I_{m,v},v\in V_{m},m\in M. \end{array}$$

where *$R_{-m,v,i}=\left[r_{m,v,1},\cdots ,r_{m,v,i-1},r_{-m,v,i},r_{m,v,i+1},\cdots ,r_{M,v,I}\right]$*,

*$r_{-m,v,i}=\left[r_{m,v,1},\cdots ,r_{m,v,i-1},r_{m,v,i+1},\cdots ,r_{m,v,I}\right]$*.

It is noted that the only difference between Problem 3 and Problem 4 is that the objective function differs by a negative sign.

**Proof.**  First, we compute the second-order derivatives of $U^{{}^{\prime }}$ in terms of $r_{m,i}$ and have

$$\begin{array}{cc} \; & \frac{\partial^{2}U^{{}^{\prime }}}{\partial r_{m,v,i}^{2}}=-\frac{2s\psi w_{m,v,i}}{Br_{m,v,i}^{3}}\\ \; & +w_{m,v,i}\eta \left[\left(1-\psi \right)\left(\frac{4\eta^{2}}{{\left(1-\rho_{m,v,i}\right)}^{3}}+\frac{\eta^{2}}{{\left(\rho_{m,v,i}-1\right)}^{3}}-\frac{4\eta^{2}}{\rho_{m,v,i}}\right)\right]\\ \; & -\xi_{m,v,i}{\left(\ln2\right)}^{2}2^{r_{m,v,i}} \end{array}$$

where $w_{m,v,i}=\theta_{m,v,i}\sum_{a=i}^{I}N_{m,v}q_{m,v,a}-\theta_{m,v,i+1}\sum_{b=i+1}^{I_{m,v}}N_{m,v}q_{m,v,b},i<I_{m,v},m\in M$,

$w_{m,v,i}=N_{m,v}q_{m,v,i}\theta_{m,v,i},i=I_{m,v},m\in M$

and $\xi_{m,v,i}=\frac{\sum_{m\in M}\sum_{v\in V_{m}}\sum_{j\in N_{m,v},h_{m,v,j}\geq h_{m,v,n}}N_{m,v}q_{m,v,j}d_{m,v,j}h_{m,v,j}}{h_{m,v,i}}$.

When $r_{m,v,i}<\frac{1}{\eta }$ and $R_{-m,v,i}$ is fixed, $U^{{}^{\prime }}$ is strictly convex on $r_{m,v,i},\forall v\in V_{m},\forall m\in M$. Therefore, $U^{{}^{\prime }}$ is a block multi-convex function in terms of r [41]. Second, constraints (**Problem 4 C1**) is obviously the set of linear constraints. Thus, when $R_{-m,v,i}$ is fixed, **Problem 4** is a convex problem in terms of $r_{m,v,i}$.    □

Referring to [41], a block coordinate descent (BCD) method is used to solve **Problem 4** and get $r^{*}$. When the monotonicity cannot be met, we can utilize the infeasible sub-sequence replacing algorithm [42] to meet the monotonicity. Finally, we derive $p^{*}$ by substituting $r^{*}$ into Equation (22).

In this subsection, we lay the groundwork for an in-depth exploration of nuanced contract formulation by anchoring our approach to an established analytical methodology. Next, we move from the traditional paradigm to the AI paradigm.

### 4.2. AI-Generated Contract Design

The presence of distinct variables among users and the intricate nature of the wireless environment contribute to the intricacy of the contract structure, thus requiring periodic modifications using established mathematical methodologies. Therefore, we use a methodology based on diffusion models to tackle the above dilemmas.

**Figure 2:**
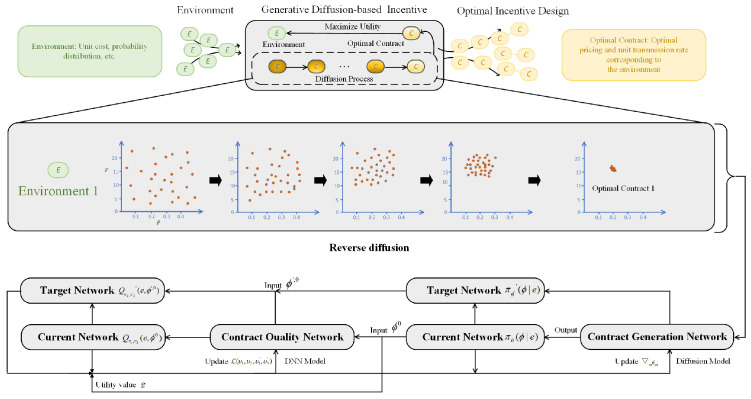
Process of producing an AI-generated contract.

In order to build a contract design, our AI-generated contract algorithm uses a denoising technique. It also incorporates exploration noise to gain experience in the exploration process. As shown in Figure 2, the processes taken by the algorithm to produce an AI-generated contract are as follows:

The diffusion-generated contract algorithm is designed to create optimal contracts through a blend of denoising methods, exploration noise, and policy optimization. It starts by defining a vector $e=\left\{h,N,q,c,\zeta ,\theta \right\}$ that encapsulates the current status of the contract service sector. The latent policy space, represented as $\pi_{\omega }\left(\phi |e\right)$, is explored using a conditional diffusion model. This exploration seeks the best contract $\phi^{0}$ based on the given state. Gaussian noise is introduced to $\phi^{K}$ and then iteratively denoised to yield $\phi^{0}$ to $\phi^{K}$, allowing for precise contract adjustments. The specific mathematical form is as follows:

(25) $$\pi_{\omega }\left(\phi |e\right)=P_{\omega }\left(\phi^{0:K}|e\right)=K\left(\phi^{K};0,I\right)\prod_{k=1}^{K}p_{\omega }\left(\phi^{k-1}|\phi^{k},e\right)$$

where $P_{\omega }\left(\phi^{k-1}|\phi^{k},e\right)$ can be modeled as a Gaussian distribution. This is specifically represented as: $K\left(\phi^{k-1};\mu_{\omega }\left(\phi^{k},e,k\right),\Sigma_{\omega }\left(\phi^{k},e,k\right)\right)$.

According to [43], the covariance matrix $\Sigma_{\omega }\left(\phi^{k},e,k\right)$ is defined as $\beta_{k}I$, and the mean $\mu_{\omega }\left(\phi^{k},e,k\right)$ constructed as $\frac{1}{\sqrt{\alpha_{k}}}\left(\phi^{k}-\frac{\beta_{k}}{\sqrt{1-{\bar{\alpha }}_{k}}}\varepsilon_{\omega }\left(\phi^{k},e,k\right)\right)$. The training phase begins with setting key parameters such as batch size, exploration noise, and number of diffusion steps *K*. The contract generation network $\epsilon_{\omega }$ with weights ω and contract quality network $Q_{\upsilon }$ with weights υ are initialized. According to [13], using double Q-learning, the best contract generation policy may be derived by minimizing the loss function L(ω):

(26) $$\pi =\underset{\pi_{\omega }}{\arg\min}L\left(\omega \right)=-E_{\phi^{0}∼\pi_{\omega }}\left[Q_{v}\left(e,\phi^{0}\right)\right]$$

where $\pi_{\omega }$ is the contract design policy. We construct two networks, namely $Q_{\upsilon_{1}}$ and $Q_{\upsilon_{2}}$, with target networks, namely $Q_{\upsilon_{1}^{{}^{\prime }}}$ and $Q_{\upsilon_{2}^{{}^{\prime }}}$, to learn the contract quality network $Q_{v}$ in a conventional way, minimizing the Bellman operator with the double Q-learning technique [44]. We then optimize $\upsilon_{i}$ for i={1,2} by minimizing the objective

(27) $$E_{\phi_{t+1}^{0}∼\pi_{\omega^{\prime }}}\left[||\left(r\left(e,\phi_{t}\right)+\gamma \min_{i=1,2}Q_{\upsilon_{i}^{\prime }}\left(e,\phi_{t+1}^{0}\right)\right)-Q_{\upsilon_{i}}\left(e,\phi_{t}\right)|{|}^{2}\right]$$

where $\pi_{\omega^{\prime }}$ is the target contract generation network. In each iteration, the environment’s state e is observed, and actions are generated by denoising $\phi^{t-1}$ and adding exploration noise. Rewards are determined by the utility function, updating the replay buffer. Critic networks are improved using policy gradient and loss calculations, and the target networks are also updated to enhance performance over time. The final stage centers on crafting the best contract. Trained networks and environmental states e are employed to deduce the contract. Gaussian noise is denoised using reverse diffusion to obtain an optimal contract $\pi_{\omega }\left(\phi |e\right)$, ensuring efficient resource allocation and maximizing utility based on the current conditions. Overall, the AI-generated contract algorithm seamlessly combines policy optimization and denoising methods to progressively refine contract representations, optimizing resource allocation and utility even under changing environmental circumstances.

The basic elements of the AI algorithm used to generate the contract are contained in the pseudocode provided. We will provide a brief review of the text for consistency.

- Training Phase: focuses on training AI-generated contracts. It initializes key components such as replay buffer, contract generation and quality networks, and target networks. The main loop iterates through episodes and steps, where the agent interacts with the environment, generates contracts, and learns to optimize contract quality. The training process involves computing rewards, updating networks, and performing target network updates, which are core elements of reinforcement learning (RL).
- Looping Pseudo-code: indicates the inference phase. In this phase, the trained AI-generated contract is used to generate the optimal contract design based on the given environment vectors. It utilizes the $\epsilon_{\omega }$ network, which has learned from the training phase to produce effective contracts.

In general, the pseudo-code provides a systematic description of the algorithm. This is consistent with RL frameworks that are often used to train agents to perform complex decision-making tasks such as contract generation. In addition, the parameters of the resource allocation scheme generated by Algorithm 1 during the process are shown in Table 3 and Table 4.
**Algorithm 1** The algorithm for diffusion-generated contract.1:**Training Phase:**2:Input hyper-parameters: diffusion step *H*, batch size $H_{b}$, discount factor ω, soft target update parameter τ, exploration noise.3:Initialize replay buffer *R*, contract generation network, target contract generation network, contract quality network, and target contract quality network.4:**for** Episode = 1 to Max episode **do**5:    Initialize a random process K for contract design exploration6:    **for** Step = 1 to Max step **do**7:        Observe the current environment $e_{t}$8:        Based on $\phi^{k-1}|\phi^{k}=\frac{\phi^{k}}{\sqrt{\alpha_{k}}}-\frac{\beta_{k}}{\sqrt{\alpha_{k}\left(1-{\bar{\alpha }}_{k}\right)}}\varepsilon_{\omega }\left(\phi^{k},e,k\right)+\sqrt{\beta_{k}}\varepsilon$, we use $\epsilon_{\omega }$ to denoise Gaussian noise $\phi_{t}^{K}$ and generate contract design $\phi_{t}^{0}$.9:        After generating contract policy $\phi_{t}^{0}$ and obtain the reward the following formulae
$$\begin{array}{ll} g_{t} & =U_{\mathrm{sp},t}+\sum_{m\in M}\sum_{i\in I}c\left[p_{m,i}-\theta_{m,i}g\left(r_{m,i}\right)\right]\\ & +\sum_{m\in M}\sum_{i,j\in I,i\neq j}P\left[\theta_{m,i}g\left(r_{m,i}\right)-p_{m,i}-\theta_{m,i}g\left(r_{m,j}\right)+p_{m,j}\right] \end{array}$$
where P(·) is a penalty function. It implements a certain penalty when the constraint is not satisfied.10:        The record $\left(e_{t},\phi_{t}^{0}\right)$ is stored in the replay buffer *R*11:        Sample a random minibatch of $H_{b}$ records $\left(e_{k},\phi_{k}^{0},g_{k}\right)$ from *R*12:        Set $y_{k}=g_{k}+\gamma Q_{\upsilon^{{}^{\prime }}}^{{}^{\prime }}\left(e_{k},\phi_{t}^{{}^{\prime }0}\right)$, where $\phi_{t}^{{}^{\prime }0}$ is obtained using $\epsilon_{\omega^{{}^{\prime }}}^{{}^{\prime }}$13:        Minimize $L=\frac{1}{H_{b}}\sum_{k}\left(y_{k}-Q_{\upsilon }\left(e_{k},\phi_{t}\right)\right)$ and update the contract quality network.14:        Computing the policy gradient ${▽}_{\omega }\epsilon_{\omega }\approx \frac{1}{H_{b}}\sum_{k}{▽}_{\phi^{0}}Q_{\upsilon }\left(e,\phi^{0}\right){|}_{e=e_{k}}{▽}_{\omega }\epsilon_{\omega }|e_{k}$ and use the policy gradient to update the contract generation network.15:        Update the target networks: $\omega^{{}^{\prime }}\leftarrow \tau \omega +\left(1-\tau \right)\omega^{{}^{\prime }}$ and $\upsilon^{{}^{\prime }}\leftarrow \tau \upsilon +\left(1-\tau \right)\upsilon^{{}^{\prime }}$16:    **end for**17:**end for**18:Output the trained contract generation network $\epsilon_{\omega }$19:InferencePhase:20:Input the environment vector e21:Based on $\phi^{k-1}|\phi^{k}=\frac{\phi^{k}}{\sqrt{\alpha_{k}}}-\frac{\beta_{k}}{\sqrt{\alpha_{k}\left(1-{\bar{\alpha }}_{k}\right)}}\varepsilon_{\omega }\left(\phi^{k},e,k\right)+\sqrt{\beta_{k}}\varepsilon$, use $\epsilon_{\omega }$ to denoise Gaussian noise and generate the optimal contract design $\phi^{0}$.22:The optimal contract design $\phi^{0}$

In this subsection, the evolution of contracting strategies is demonstrated through the convergence of classical principles with cutting-edge AI techniques. However, there is still a need to discuss the implications of real-world scenarios. The practicalities and nuances of contract deployment are explored below.

### 4.3. Deployment of Contracts

In the intricate framework of mobile edge networks, the precise location of MTs is of paramount importance. The effectiveness of the SP is intrinsically linked to the assurance that each MT carefully selects an optimal contract item that is a good fit for its specific type. Intriguingly, the positioning of the microbase station, which hosts the MEC server, is static, underscoring its intransigent nature. Given this inflexibility, an optimal strategy pivots towards modulating a more adaptable entity: the UAV entrusted with the MEC server. By optimizing the spatial coordinates of the UAV, we can generate precision in the contract item delineation process. Upon the ratification of the contract selection across the MT spectrum, two salient parameters emerge into the limelight: the transmission rate inherent to each UAV and its concomitant reward. An astute observation at this juncture elucidates that the SP, armed with this newly found knowledge, discerns the precise location of each UAV. Such enlightenment precipitates a complex optimization conundrum: how to augment the SP’s utility by finessing the locational parameters of the edge nodes? This optimization enigma is elegantly captured as **Problem 5**:

**Problem** **5.** (28) $$\begin{array}{cc} \; & \;\max_{\left\{l_{m,v}\right\}}U_{\mathrm{sp}}\\ s.t. & \;0\leq l_{m,v,1},l_{m,v,2},l_{m,v,3}\geq L^{max},v\in V_{m},m\in M. \end{array}$$

where *$U_{\mathrm{sp}}=\sum_{m\in M}\sum_{v\in V_{m}}\sum_{i\in I_{m,v}}\left({\hat{p}}_{m,v,i}-{\hat{c}}_{1}^{m,v}d_{m,v,i}\left(\hat{r}\right)-{\hat{c}}_{2}^{m,v}{\hat{\eta }}_{m,v}{\hat{\kappa }}_{m,v}{\hat{f}}_{m,v}^{2}\right)$*. We also define *$d_{m,v,i}\left(\hat{r}\right)=\frac{\left(2^{{\hat{r}}_{m,v,i}}-1\right)\left(\zeta +\sum_{m\in M}\sum_{v\in V_{m}}\sum_{j\in N_{m,v},h_{m,v,j}\leq h_{m,v,n}}d_{m,v,j}h_{m,v,j}\right)}{h_{m,v,i}}$, $h_{m,v,n}={\left\|l_{m,v}-{\hat{l}}_{m,v,n}\right\|}^{-2}$.* And* $L_{max}$* is the maximum communication range of the edge node.

Within this mathematical formulation, $U_{\mathrm{sp}}$ epitomizes the holistic utility derived by the SP. This metric is an amalgamation of a panoply of variables, encompassing the fiscal dynamics associated with diverse transmission rates and a confluence of incentives vis-à-vis the spatial dynamics of MTs.

Nevertheless, the inherent nonconvexity of **Problem 5** augments the problem’s complexity. To navigate this intricate maze, our strategic pivot leans towards the meta-heuristic paradigm, notably harnessing the prowess of the improved differential evolution (DE) algorithm. We propose finding optimal location using differential evolution algorithm, and the specific process is represented by Algorithm 2.
**Algorithm 2** Finding optimal location using differential evolution algorithm.1:**Input:** Population: *Z*; Dimension: *W*; Generation *K*2:**Output:** The best vector $L=\left\{l_{m,v}^{*},v\in V_{m},m\in M\right\}$3:Initialize the number of iterations k=1;4:**for** z=1 to *Z* **do**5:    **for** w=1 to *W* **do**6:        $L_{k,z}^{w}=L_{\min}^{w}+\mathrm{rand}\left(0,1\right)\left(L_{\max}^{w}-L_{\min}^{w}\right)$7:    **end for**8:**end for**9:**while** $|U_{\mathrm{sp}}\left(L^{*}\right)|\geq \epsilon$ or k≤K **do**10:    **for** z=1 to *Z* **do**11:        Mutation and Crossover12:        **for** i=1 to *I* **do**13:           $\varrho_{k,z}^{w}=\mathrm{Mutation}\left(L_{k,z}^{w}\right)$;14:           $\vartheta_{k,z}^{j}=\mathrm{Crossover}\left(L_{k,z}^{w},\varrho_{k,z}^{w}\right)$;15:        **end for**16:        Greedy and Selection17:        **if** $U_{\mathrm{sp}}\left(\vartheta_{k,z}\right)<U_{\mathrm{sp}}\left(L_{k,z}\right)$ **then**18:           $L_{z}\leftarrow \vartheta_{z}$19:           **if** $U_{\mathrm{sp}}\left(L_{k,z}\right)<U_{\mathrm{sp}}\left(L^{*}\right)$ **then**20:               $L^{*}\leftarrow L_{k,z}$21:           **end if**22:        **end if**23:        $L_{z}\leftarrow L_{k,z}$24:    **end for**25:    k←k+126:**end while**27:**Return** $L=l_{m,v}^{*},v\in V_{m},m\in M$

## 5. Simulation Results

We perform simulations in MATLAB to verify that the proposed incentive mechanism is effective. The numerical results show that the proposed incentive mechanism has a great advantage in improving the utility of the system when compared with other benchmark incentives. We consider that the probability of each type is uniform in (0,20]. Referring to [45,46,47,48], other parameters are summarized in Table 5.

### 5.1. Evaluation of the Proposed Contract Design under Certain Environment

#### 5.1.1. Efficiency of Contract Design

**Figure 3:**
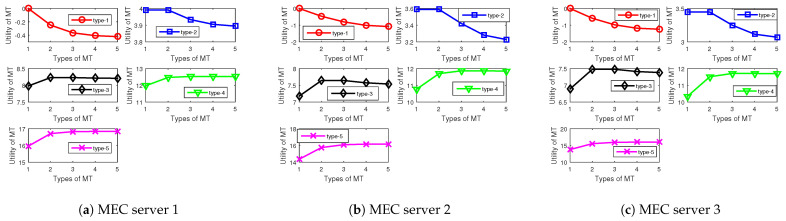
Types of MT versus utilities of MTs under different MEC servers.

In Figure 3, we evaluate the IR and the IC conditions of our proposed algorithm. Figure 3 shows the utilities of type-1, type-2, type-3, type-4, and type-5 MTs when selecting all the contracts $(P_{m,i},r_{m,i}),i\in I,m\in M$ offered by MEC servers. For example, for type 1, it can be seen that each MT can maximize its utility when selecting the contract that fits its own types, which means that the IC constraints are satisfied. Furthermore, each type of MTs receives a positive utility value when selecting the contract that fits their types, which suggests that the IR constraints are satisfied. Therefore, after choosing the best contract that is designed for its own type, the types of the MTs will be revealed to the MEC server. Therefore, by applying the proposed scheme, each MEC server is able to be aware of the exact types of the MTs, which means that the asymmetric information between the MEC server and the MTs is overcome. Moreover, the utilities of higher type of MTs are larger than those of lower type of MTs.

#### 5.1.2. Impact of Interference on System Performance

**Figure 4:**
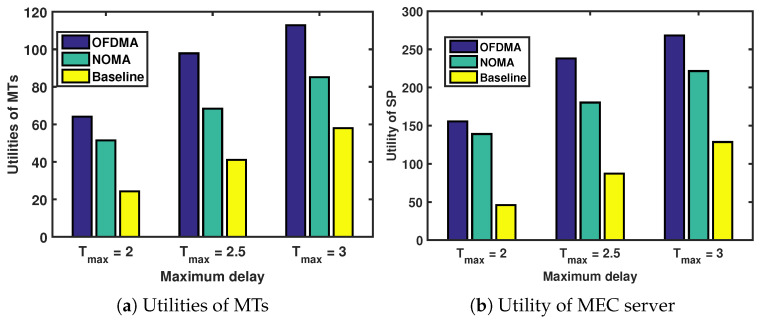
Utilities of MTs and MEC server versus number of MT types under different interference strategies.

Figure 4 shows the performance system versus maximum delay under different interference strategies. Details of these interference strategies are listed as follows:

- OFDMA: Each MT in cross-layer and co-layer adopts orthogonal frequency-division multiple access (OFDMA) to offload computation tasks. OFDMA optimizes bandwidth via orthogonal subcarriers, ensuring interference-free transmission. Users on different subcarriers achieve mutual orthogonality, enhancing communication efficiency.
- NOMA: In both cross-layer and co-layer scenarios, each MT employs NOMA as a means to offload computational chores. NOMA permits multiple users to share the same subchannel, boosting spectral efficiency and ensuring rapid transmission. Unlike OFDMA, NOMA has less of the near–far effect and works best in dynamic link states, maintaining a strong rate performance even when there are problems with multi-access interference.
- Baseline: Each MT in both cross-layer and co-layer settings shares the same channel for computational task offloading.

The utilities of the SP and the MTs increase with the maximum delay, as shown in Figure 4a and Figure 4b, respectively. Additionally, we analyze the utilities of the SP and the MTs under different interference strategies when the maximum delay is fixed.

#### 5.1.3. Performance Comparison

We compare the proposed contract-based incentive mechanism under asymmetric information (CA) with other incentive mechanisms, including the Stackelberg game-based incentive mechanism under asymmetric information (SA) [33]. For the SA mechanism, the utilities of MTs and the SP are as follows:

(29) $$\begin{array}{c} u_{m,i}^{\mathrm{SA}}=\theta_{m,i}g\left(d_{m,i}^{\mathrm{SA}}\right)-d_{m,i}^{\mathrm{SA}}p_{m,i}^{\mathrm{SA}}. \end{array}$$

and

(30) $$\begin{array}{c} U^{\mathrm{SA}}=\sum_{m\in M}\sum_{i\in I_{m}}N_{m}q_{m,i}U_{m,i}^{\mathrm{SA}} \end{array}$$

where $U_{m,i}=d_{m,i}^{\mathrm{SA}}p_{m,i}^{\mathrm{SA}}-c_{1}d_{m,i}^{\mathrm{SA}}-c_{2}\eta \kappa f^{2}$.

**Figure 5:**
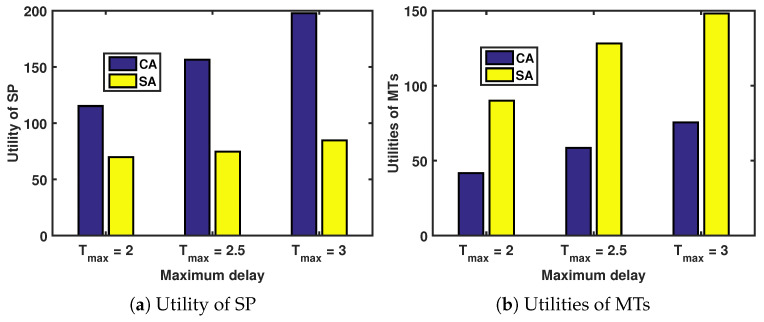
Utilities of MTs and the SP versus maximum delay under different incentives.

Figure 5 depicts the relationship between the utilities of the MTs (and the SP) and maximum delay under different incentive mechanisms.

The utilities of the SP and the MTs increase with the maximum delay, as shown in Figure 5a and Figure 5b, respectively. Additionally, we analyze the utilities of the SP and the MTs under different incentive mechanisms when the maximum delay is fixed. Since the research objective is to maximize the utility of SP, it is necessary to ensure that the SP utility is maximized when the MT utility is non-negative.

In Figure 5a, the CA approach achieves the best performance among the two approaches. This is because the CA approach aims to collect as much revenue from the MTs as possible while satisfying both the IR and IC constraints, leaving only a small share of revenue for the MTs. In contrast, the SG strategy aims to maximize the combined utility of the SP and the MTs, allowing for more revenue to be allocated to the MTs. In Figure 5b, the CA approach provides better utilities for the MTs than the SA approach. This is due to the same reasons mentioned in Figure 5a.

#### 5.1.4. Impact of System Parameters on System Performance

**Figure 6:**
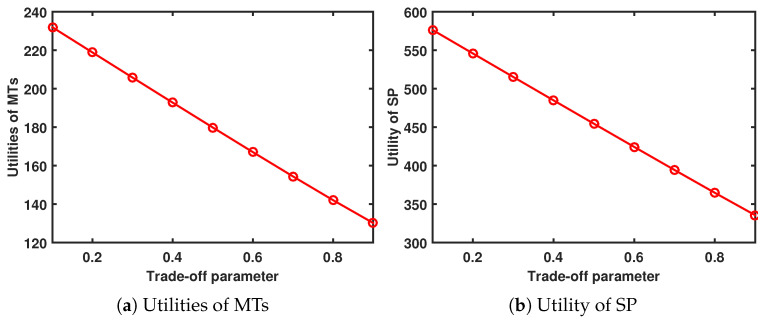
Performance system versus trade-off parameter.

When $T^{\max}=4s$ and $A^{\max}=13$, Figure 6 shows the performance system versus trade-off parameter. As ψ increases, the utility of the MTs and SP decreases.

**Figure 7:**
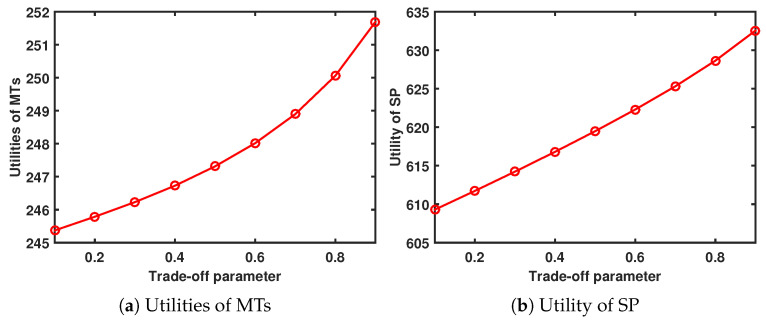
Performance system versus trade-off parameter.

When $T^{\max}=8s$ and $A^{\max}=13$, Figure 7 shows the performance system versus the trade-off parameter. As ψ increases, the utility of the MTs and SP increases.

**Figure 8:**
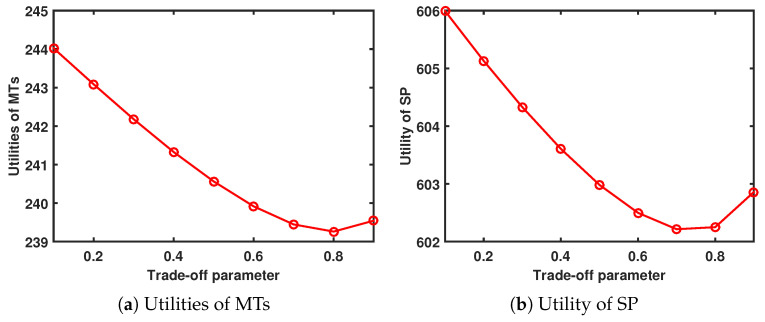
Performance system versus trade-off parameter.

When $T^{\max}=7.6s$ and $A^{\max}=13$, Figure 8 shows the performance system versus trade-off parameter. As ψ increases, the utility of the MTs and SP first decreases and then decreases.

**Figure 9:**
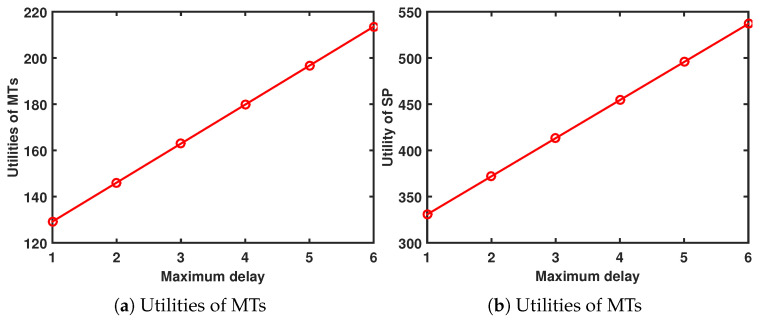
Performance system versus maximum delay.

When ψ=0.5 and $A^{\max}=13$, Figure 9 shows the performance system versus maximum delay. As $T^{\max}$ increases, the utility of the MTs and SP increases. The increase in $T^{\max}$ increases the gain for the MTs, so the MTs are willing to pay more rewards for the SP, which means that the utility of the SP also increases.

**Figure 10:**
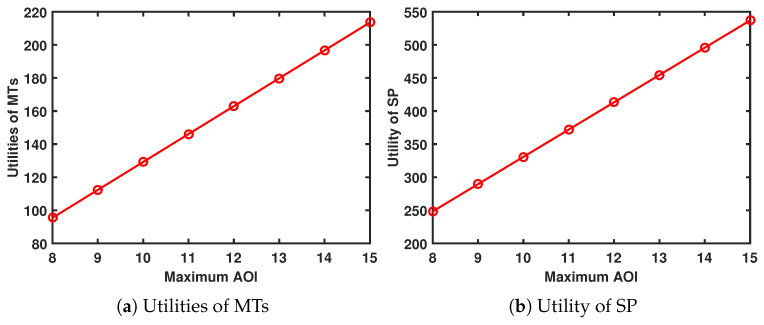
Performance system versus maximum AoI.

When ψ=0.5 and $T^{\max}=4s$, Figure 10 illustrates the performance system in relation to the maximum AoI. As the maximum AoI increases, the utilities of both the MTs and the SP increase. The MTs gain more when the maximum AoI is higher, so they are willing to offer more rewards to the SP, thus increasing the SP’s utility.

### 5.2. Evaluation of the Proposed Generative AI-Aided Contract Design under Uncertain Environment

**Figure 11:**
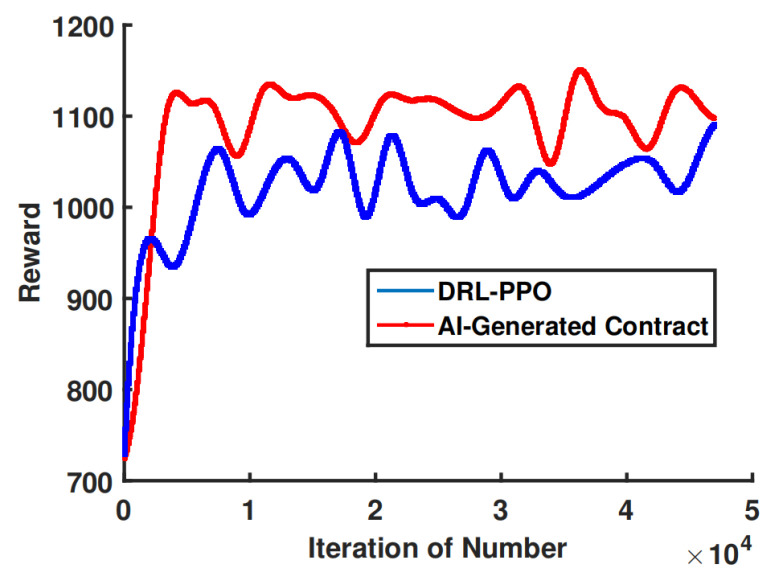
Training process.

We conducted a comparative analysis of our suggested artificial intelligence (AI)-generated contract algorithm and two traditional DRL algorithms, namely SAC and proximal PPO. The training process, as depicted in Figure 11, demonstrates that the PPO algorithm requires a greater number of iteration steps in order to achieve convergence. Although the SAC method exhibits a relatively rapid stabilization at a higher reward level, it is important to note that both its convergence rate and final reward value are comparatively worse when compared to the AI-generated contract approach. This phenomenon can be attributed to two factors:

- Our algorithm produces higher-quality samples by utilizing diffusion models and fine-tuning multiple times. By setting the diffusion step to 10 and gradually adjusting the model’s output with each fine tuning, the impact of uncertainty and noise is reduced, resulting in improved sampling accuracy.
- Our approach is better able to handle long-term dependencies. In contrast to traditional neural network generation models that solely take into account the input at the present time step, the diffusion model undergoes numerous fine-tuning iterations to produce samples including a greater number of time steps. This phenomenon leads to an enhanced capacity for long-term reliance processing.

**Figure 12:**
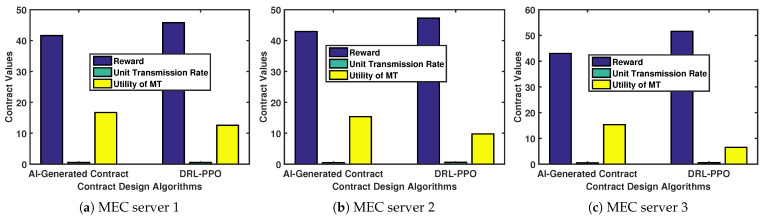
Contract design algorithms.

Figure 12 shows the comparison of the trained models’ capacity for optimal contract design. For type-3 MTs, when given a particular environment state, the AI-generated contract algorithm can generate a contract design that yields a higher utility than the utility obtained by the DRL-PPO.

### 5.3. Contract Deployment Optimal Design

**Figure 13:**
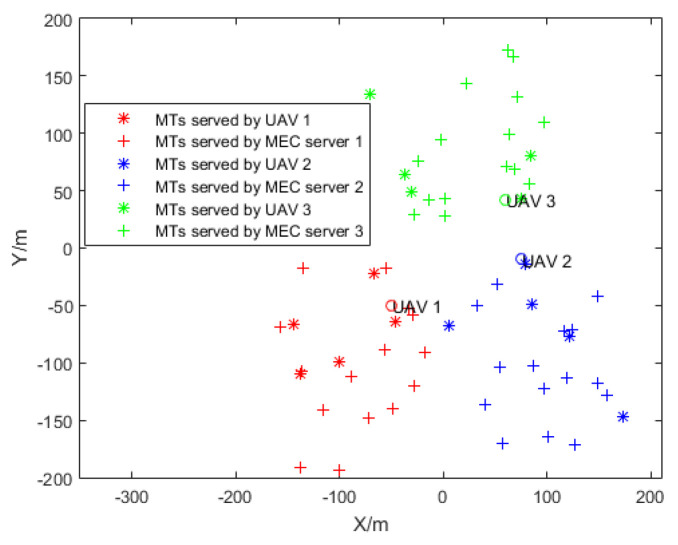
Optimal location of UVAs in different MEC servers.

**Figure 14:**
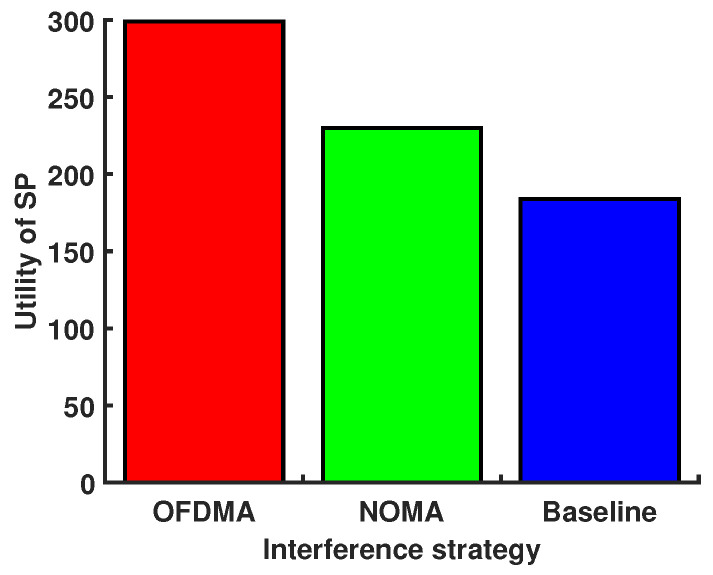
Utility of the SP versus different interference strategies under the optimal location of UVAs.

We consider a scenario in which there are three MEC servers, and every MEC server located within a 100 m radius has its own UAV. We set the location of three MEC severs as (−100 m, −100 m), (100 m, −100 m), and (0, 100 m). Then, 20 users are randomly generated within the 100 m radius of each MEC server. Fifteen of these users are served by each MEC server, and five are served by the UAV. We show in Figure 13 that the service delivery of MT is enhanced by optimizing the location coordinates of the UAVs, the result of which leads to an optimal contract deployment design. Figure 14 shows the utility of the SP versus different interference strategies under the optimal location of UVAs. Since there is no interference in the OFDMA strategy, the SP allows MTs to use the lowest power to achieve a transmission rate that meets the delay and AOI requirements, which reduces the SP cost and maximizes the utility of the SP. Interference with the baseline strategy causes MTs to use more power to meet latency and AOI requirements, increasing the SP cost and minimizing the utility of the SP.

## 6. Conclusions

We suggest implementing an incentive system that comprises two distinct stages: contract design and deployment. During the contract design phase, we create a mechanism using contract theory. It optimizes the utility of the SP while satisfying the IR and IC requirements of the mobile customer. We use the block coordinate descent method to discover the most favorable contract and introduce a new strategy based on the generative diffusion model to improve the efficiency of finding the best contract. During the contract deployment phase, the effectiveness of SP contract deployment is enhanced by optimizing the positioning of UAVs. To do this, a refined differential evolutionary algorithm is introduced to determine the most optimum site for the UAVs. The rigorous empirical evaluation shows that under incentive-consistent conditions, our approach has improved over the baseline approach (each MT in both cross-layer and co-layer settings shares the same channel for computational task offloading) by nearly 100%. When NOMA is employed, the incentive methods we use nearly double the SP efficiency on average.

In the next step of our research, we will discuss how UAV trajectories and UAV speeds affect resource allocation strategies.

## Figures and Tables

**Table 1:** Optimization of each metric by computational tasks in MEC.

Ref.	Optimization Perspective	Energy Consumption	Delay	AoI	UAV	Interference	
						**OFDMA**	**NOMA**
[19]	System	🗸		🗸			
[20]	System			🗸		🗸	
[25]	System			🗸	🗸		
[21]	System			🗸			
[17,18]	System		🗸	🗸			
[22]	System	🗸	🗸	🗸			
[26]	System		🗸				🗸
[27]	System			🗸			🗸
[31]	Incentive		🗸	🗸			
[28,29]	Incentive			🗸		🗸	🗸
[23,24]	Incentive	🗸		🗸	🗸		
[32,33]	Incentive			🗸			

**Table 2:** Key notations.

Symbol	Description
m∈M	Set of MEC servers
$n_{m,v}\in N_{m,v}$	Set of MTs
$v_{m}\in V_{m}$	Set of UAVs
$\mu_{m,v,n}^{t}$	Service rate of the transmission queue
$\mu_{m,v,n}^{s}$	Service rate of the MEC computation queue
$\rho_{m,v,n}<1$	Queue utilization of device $n_{m}$ in MEC server *m*
δ(Mcycles)	CPU cycles
*s*(MBits)	Data packet size
$f_{m,v,n}$	MEC server’s computational capacity
$h_{m,v,n}$	Channel power gain between MEC server and MT
φ	Channel power at the reference distance
$l_{m,v}$	Location of the MEC server *m* or UAV $v_{m}$
$l_{m,v,n}$	Location of MT $n_{m,v}$
$d_{m,v,n}$	Transmission power of MT
*B*	Bandwidth
ζ	Power spectral density of Gaussian white noise
$R_{m,n}$	Transmission rate of task offloading
$A_{m,v,n}$	Average AoI of MT $n_{m}$
$T_{m,v,n}$	Average latency of service
$A_{max}$	Maximum tolerated AoI
ψ	Trade-off parameter between the saved delay and saved AoI
$g(d_{m,v})$	Gain of MT when offloading tasks
$p_{m,v,n}$	Cost of SP
$\theta_{m,v,n}$	Satisfaction gained from the saved delay performance
$u_{m,v}$	Utility of MT
$I_{m,v}$	Category of MT
$q_{m,v,i}$	Probability distribution of category
$c_{1}^{m,v}d_{m,v,i}$	Unit costs paid by SP for offloading computing tasks
$c_{2}^{m,v}\eta \kappa_{m,v}f_{m,v}^{2}$	Computational cost of completing the task
$\kappa_{m,v}$	Effective switching capacitance
e	State spaces
$\pi_{\omega }\left(\phi |e\right)$	Latent policy space
$\phi^{0}$	Optimal contract for given state
$\phi^{K}$	Gaussian noise
L(ω)	Loss function

**Table 3:** Algorithm network parameters.

Networks	Layer	Activation	Units
Actor	SinusoidalPosEmb FullyConnect FullyConnect Concatenation FullyConnect FullyConnect FullyConnect	- Tanh - - Tanh Tanh Tanh	16 32 16 - 256 256 12
Critic	FullyConnect FullyConnect FullyConnect FullyConnect	Mish Mish Mish -	256 256 256 1

**Table 4:** Algorithm training parameters.

Parameter	Value
Learning rate of the actor network	$10^{-6}$
Learning rate of the critic networks	$10^{-6}$
Temperature of action entropy regularization	0.05
Weight of soft update	0.005
Batch size	512
Weight decay	$10^{-4}$
Discount factor to accumulate rewards	0.95
Diffusion steps for the diffusion model	5
Maximum capacity of the replay buffer	$10^{6}$
Total number of training steps	$10^{5}$
Number of collected transitions per training step	1000

**Table 5:** Parameter settings in the simulation.

Parameter	Setting
Number of MEC servers	M=3
Number of UAVs	V=3
Number of types	$N_{1}=N_{2}=N_{3}=5$
Effective switched capacitance	$\kappa =10^{-28}$
Number of CPU cycles executing one bit	η=β∈(0,10] cycles/bit
Maximum tolerance time	$T_{\max}\in \left(0,6\right]$ s
Bandwidth	$B=10^{6}$ Bits
Computational capability	$f=10^{10}$ cycles/bit
Size of computation task	s=10 KB
Others	$c_{1}=c_{2}=1.5$, $\delta =10^{9}$

## Data Availability

Data are contained within the article.
